# 18FDG PET Assessment of Therapeutic Response in Patients with Advanced or Metastatic Melanoma Treated with First-Line Immune Checkpoint Inhibitors

**DOI:** 10.3390/cancers14133190

**Published:** 2022-06-29

**Authors:** Alexia Rivas, Julie Delyon, Antoine Martineau, Estelle Blanc, Clara Allayous, Laetitia Da Meda, Pascal Merlet, Céleste Lebbé, Barouyr Baroudjian, Laetitia Vercellino

**Affiliations:** 1 Department of Nuclear Medicine, AP-HP Saint-Louis Hospital, F-75010 Paris, France; alexia.rivas@neuf.fr (A.R.); antoine.martineau@aphp.fr (A.M.); estelleblancautran@gmail.com (E.B.); pascal.merlet@aphp.fr (P.M.); 2 Department of Dermatology, AP-HP Saint-Louis Hospital, F-75010 Paris, France; julie.delyon@aphp.fr (J.D.); clara.allayous@aphp.fr (C.A.); laetitia.da-meda@aphp.fr (L.D.M.); celeste.lebbe@aphp.fr (C.L.); barouyr.baroudjian@aphp.fr (B.B.); 3 Université Paris Cité, INSERM U976, F-75010 Paris, France; 4 Université Paris Cité, F-75006 Paris, France; 5 Université Paris Cité, INSERM UMR_S942, F-75006 Paris, France

**Keywords:** FDG PET, melanoma, immunotherapy, therapeutic response, immune checkpoint inhibitors

## Abstract

**Simple Summary:**

In a retrospective study of patients with advanced or metastatic melanoma treated with first-line immune checkpoint inhibitors, we investigated the value of metabolic criteria, PERCIST 5 (criteria used for conventional chemotherapy), and imPERCIST5 (criteria adapted for immunotherapy therapeutic evaluation). Responding patients according to both criteria had better overall survival than that of not-responding patients, with a 2 years OS of 91% versus 39%, respectively. Combining different approaches to assess response could help improve the confidence in the test aiming at evaluating the response to immunotherapy.

**Abstract:**

Background: Immune checkpoint inhibitors (ICI) are currently the first-line treatment for patients with metastatic melanoma. We investigated the value of positron emission tomography (PET) response criteria to assess the therapeutic response to first-line ICI in this clinical context and explore the potential contribution of total tumor metabolic volume (TMTV) analysis. Methods: We conducted a retrospective study in patients treated with first-line ICI for advanced or metastatic melanoma, with 18F-FDG PET/CT performed at baseline and 3 months after starting treatment. Patients’ metabolic response was classified according to PERCIST5 and imPERCIST 5 criteria. TMTV was recorded for each examination. Results: Twenty-nine patients were included. The median overall survival (OS) was 51.2 months (IQR 13.6—not reached), and the OS rate at 2 years was 58.6%. Patients classified as responders (complete and partial response) had a 90.9% 2-year OS rate versus 38.9% for non-responders (stable disease and progressive disease) (*p* = 0.03), for PERCIST5 and imPERCIST 5 criteria. The median change in metabolic volume was 9.8% (IQR −59–+140%). No significant correlation between OS and changes in TMTV was found. Conclusion: The evaluation of response to immunotherapy using metabolic imaging with PERCIST5 and imPERCIST5 was significantly associated with OS in patients with advanced or metastatic melanoma.

## 1. Introduction

Melanoma is a rare skin cancer (5% of all skin cancers) but accounts for most of its lethality [1]. Moreover, the incidence of melanoma throughout the world has been increasing for the last 50 years [2]. Until a decade ago, advanced or metastatic melanoma resulted in a poor prognosis [3]. The emergence of new therapies such as immune checkpoint inhibitors (ICI), represented by anti-PD1 and anti-CTLA4, have improved the overall survival (OS) and progression-free survival (PFS) of patients with advanced and metastatic melanoma and are now validated for their therapeutic management [4,5,6]. However, not all patients will benefit from this approach, with an overall response rate of about 40% for anti-PD1 monotherapy and long-term survival of about 35% [6,7]. The combination of nivolumab and ipilimumab improves the overall response rate to over 50%, with a trend for better OS (more than 52% of OS at 5 years with the nivolumab/ipilimumab combination versus 44% for nivolumab as monotherapy), at the cost of increased toxicities [4]. Moreover, based on their mechanism of action, the efficacy of these ICI may be delayed and result in a late clinical response or even in a transitory increase in tumor volume [8,9]. FDG PET has largely been used for staging advanced melanoma and restaging at relapse [10,11] with good diagnostic accuracy, leading to its use for therapeutic evaluation in patients receiving systemic therapy, especially ICI [12]. In addition to what has been proposed for the morphological evaluation of response to ICI, with immune-related response criteria [8], some new PET criteria have been proposed to improve the prediction of response to ICI, including the immunotherapy-modified Positron Emission Tomography Response Criteria in Solid Tumors (imPERCIST) [13,14,15,16,17]. These new criteria share the common feature that the progression classification requires more than the appearance of new lesions, contrary to the conventional PERCIST score [18]. However, their clinical utility remains controversial, and to the best of our knowledge, only one study applied the imPERCIST5 criteria to a series of patients treated with ipilimumab, and none in patients on first-line antiPD1 (or anti PD1/anti CTLA4 combination) for advanced/metastatic melanoma [16]. Moreover, FDG PET-derived metabolic volume seems to be a highly promising approach as a prognostic factor on baseline FDG PET, as well as in the follow-up FDG PET examinations [19,20,21,22,23,24]. We investigated whether these two PET/CT criteria (PERCIST5 and imPERCIST5) had prognostic value in patients receiving first-line ICI for advanced or metastatic melanoma. We also investigated the potential contribution of total metabolic tumor volume (TMTV) change for therapeutic evaluation and prognosis.

## 2. Materials and Methods

### 2.1. Patients

We conducted a retrospective monocentric study on patients from Melbase, a national prospective clinical cohort (NCT02828202) at St. Louis Hospital (Paris, France). Inclusion criteria were patients with unresectable stage III or stage IV melanoma, aged over 18 years, treated with first-line ICI (nivolumab, pembrolizumab or nivolumab + ipilimumab combination), who performed a PET/CT maximum two months prior to treatment, and PET/CT two to four months after initiating ICI, on the same PET/CT device. Exclusion criteria were patients who did not have target lesions on initial PET/CT, patients with only brain metastasis and patients with another progressive solid neoplasia or blood cancer. Main data regarding patients’ demographics and outcomes, disease characteristics and treatment details were collected. 

All patients had given informed consent, and the study was approved by the regional ethics committee (n° 12,027, 2012).

### 2.2. 18F-FDG PET/CT Protocol

All patients performed their exams on a Biograph mCT Flow 64-4R PET/CT system (Siemens Healthcare, Erlangen, Germany) of the Nuclear Medicine Department of the Saint-Louis University Hospital. Patients fasted for at least 6 h before the 18F-FDG injection. If the plasma glucose level was below 11 mmol/L, patients received an intravenous injection of 3 MBq/kg of Fluorine-18 (^18^F) fluorodeoxyglucose (FDG). After the injection, patients had a resting period of 60 +/−10 min before image acquisition. All PET/CT acquisitions were performed from the vertex to the toes without an injection of iodine contrast material. Each examination started with a CT scan for correction of attenuation and anatomical localization, immediately followed by PET acquisition. PET images were reconstructed (Matrix: 200 × 200, Voxel: 4 × 4 × 3 mm^3^) with mCT software using OP-OSEM (Ordinary Poisson Ordered Subset Expectation Maximization) and TOF and PSF corrections. Images were post-smoothed with a 4 mm-FWHM Gaussian kernel. 

### 2.3. Image Analysis

Two experienced nuclear medicine physicians (AR and LV) interpreted the images independently and blinded to clinical data, followed by a consensus for discordant results. Syngovia (Siemens) and Fiji software were used for image analysis. For every patient, the SULpeak of each target lesion and the TMTV were recorded. SUL is an estimate of the rate of glucose metabolism normalized by lean mass. The investigators placed a sphere corresponding to the volume of interest (VOI) around the target lesion. In this VOI, the software searched for the 1.0 cm^3^ sphere that included the voxels with the highest mean SUL: this SUL was reported as SULpeak. The percentage of change in the sum of lesions SULpeak between the baseline and follow-up examinations was calculated and defined as ∆SULpeak:

ΔSULpeak=(∑ SULpeak follow up PET −∑ SULpeak baseline TEP)/(∑ SULpeak baseline)×100.

The investigators then classified patients into one of four categories: complete metabolic response (CR), partial metabolic response (PR), stable metabolic disease (SD), or progressive metabolic disease (PD) according to the PERCIST 5 and imPERCIST 5 criteria as follows:

- PERCIST5 criteria (PET Response Criteria in Solid Tumors): nuclear physicians measured SULpeak on up to 5 metastatic lesions and no more than 2 per organ. The lesions could be different on baseline and follow-up PET/CT. The target lesions had to have a SULpeak greater than the reference threshold defined as follows: on the healthy liver: reference threshold = 1.5* SULmean liver +2* σSULliver; on the metastatic liver: reference threshold = 2* SULmean mediastinum +2* σSULmediastinum. CR was defined as complete resolution of 18F-FDG within measurable target lesion so that it is less than mean liver activity and indistinguishable from surrounding background blood-pool levels. PR was defined as a decrease of more than 30% in the sum of SULpeak. PD was defined as an increase of more than 30% in the sum of SULpeak or the appearance of new lesions. Finally, SD applies to patients who do not fit into the definitions of CR, PR or PD.
- imPERCIST5 criteria (modified PERCIST criteria for immunotherapy): the analysis of the 5 lesions was performed similarly to PERCIST5, with the difference that the appearance of a new lesion did not lead automatically to PD. Thus, the SULpeak of the new lesion(s) had to be included in the sum of the SULpeak of the 5 hottest tumor lesions, and PD was only defined by an increase of more than 30% in the sum of the SULpeak. New lesion(s) were included in the sum of total SULpeak if they had a SULpeak greater than pre-existing target lesions or if less than 5 target lesions were detected at baseline.

In addition, TMTV, expressed in mL, was determined for each patient. TMTV was measured using the 41% SUVmax threshold method. These measurements were obtained using the Fiji software by semi-automatic contouring. The investigators had to ensure that each semi-automatically contoured lesion corresponded to a tumor lesion, and the volume could be manually delineated if necessary.

### 2.4. Statistical Analysis

The results are given as median with their interquartile range. Concordance between the imPERCIST5 and PERCIST5 criteria was evaluated using Cohen’s kappa test. PFS and OS curves were obtained using the Kaplan–Meier method. PFS was defined as the time from treatment initiation until progression or death (from any cause). OS was defined as the time from treatment initiation until death (from any cause). For the OS and PFS analysis, the data were dichotomized into responder patients (CR and PR) and non-responder patients (SD and PD). The log-rank test was used to evaluate the differences between the Kaplan–Meier curves for each evaluation method. The Younden Index method for determining the metabolic volume variation cut-off (∆T-MTV) was applied to discriminate patients into two groups. A *p* value of 0.05 or less was considered significant.

## 3. Results

### 3.1. Patient Characteristics

**Figure 1:**
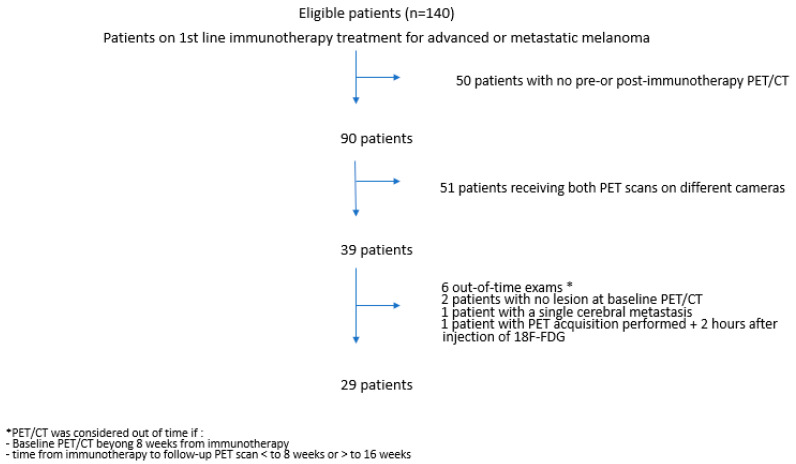
Flow chart.

Between January 2015 and May 2019, 29 patients were included in the study (Figure 1). Fifteen patients were on first-line treatment with pembrolizumab, nine with nivolumab and five with nivolumab + ipilimumab combination. Patient characteristics are summarized in Table 1. The median age at treatment initiation was 64 years (IQR 56–79). Eight patients (27%) had a high LDH level, and 24% had a BRAF-mutated melanoma. Two patients had brain metastasis before the onset of treatment.

### 3.2. PET/CT Acquisitions

The baseline PET/CT was performed at a median of 3 weeks before treatment initiation (IQR 1.3–3.7), and the follow-up PET/CT was performed at a median of 12.8 weeks after starting ICI (IQR 11.6–13.9). Image acquisition was completed on average 65 +/− 7 min after radiotracer injection. For three patients, target lesions at baseline had a SULpeak below the PERCIST5 reference threshold but were clearly distinct from the surrounding background and were retained for analysis. These lesions were all sub-centimetric and corresponded to skin nodules, lung nodules and para-rectal adenopathy.

### 3.3. Baseline PET/CT

On baseline PET/CT, 68 target lesions were analyzed for all patients, with a median of 2 target lesions per patient (IQR 1–3). The median sum of SULpeak per patient was 8.4 (IQR 4.8–16.7). The median TMTV was 5.6 mL (IQR 2–27).

### 3.4. Metabolic Response at 3 Months

**Figure 2:**
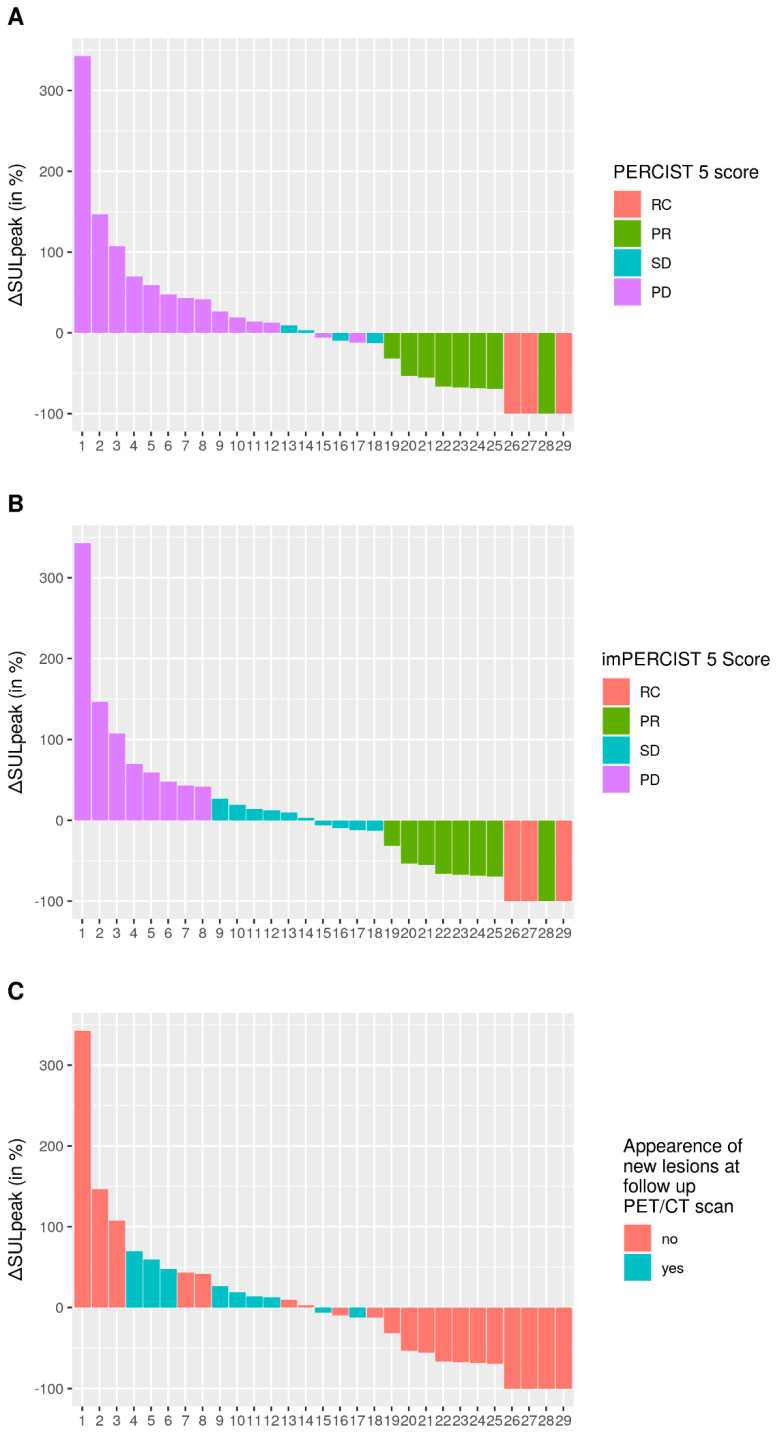
Waterfall plot of changes in SULpeak for PERCIST5 (**A**), imPERCIST5 (**B**) and according to the appearance of new lesions (**C**).

On the follow-up PET/CT, 62 target lesions were analyzed, with a median sum of SULpeak of 6.8 (IQR 1.9–14.8). Individual patients’ ∆SULpeak values are shown in Figure 2. Eleven patients (37.9%) were classified as responders according to PERCIST5 and imPERCIST5 criteria (3 CR and 8 PR, Supplementary Figure S1). Eighteen (62.1%) were classified as non-responders (14 PD and 4SD according to PERCIST 5 criteria versus 8 PD and 10 SD according to imPERCIST 5, Supplementary Figure S2) (Table 2). Overall, 14 patients (48.3%) were classified as progressive according to PERCIST5 criteria versus 8 (28%) according to imPERCIST5 criteria (Figure 2).

**Figure 3:**
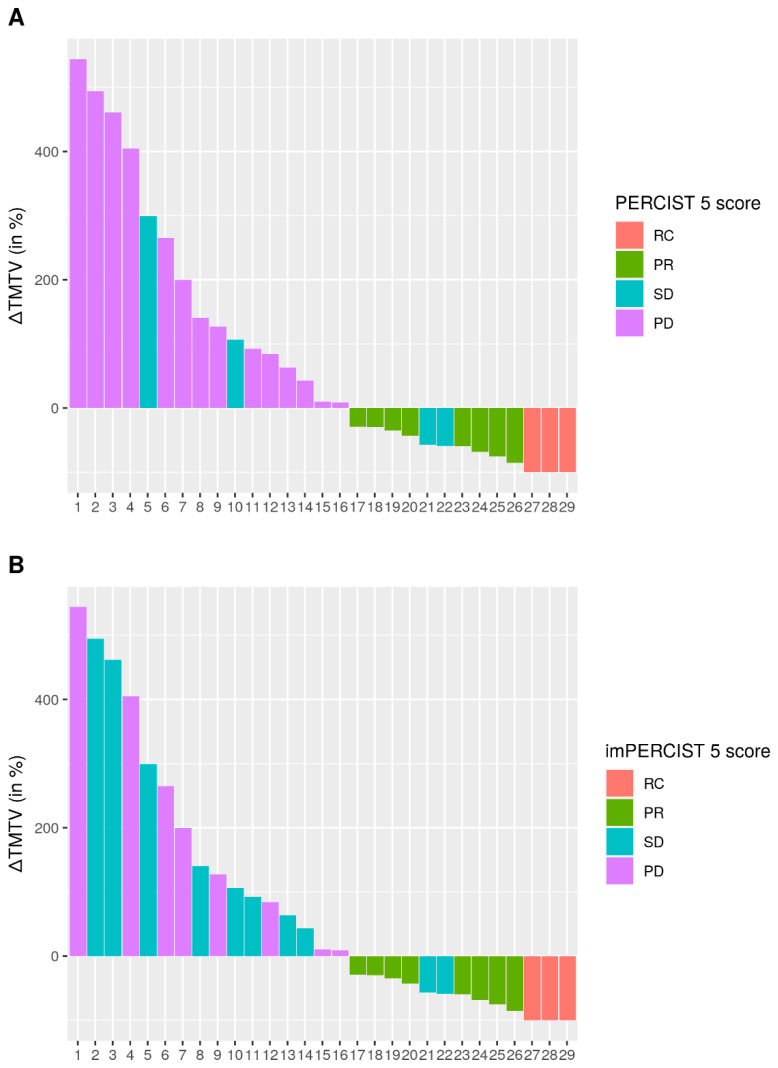
Waterfall plot of changes in total metabolic tumor volume (∆TMTV) for PERCIST5 (**A**) and imPERCIST5 (**B**).

The median follow-up TMTV was 8.6 mL (IQR 1.4–22.4). The median change in TMTV between the two PET/CT (∆TMTV) was thus +9.8% (IQR −59–+140%). The ∆TMTV for each patient is shown in Figure 3.

The median ∆TMTV in non-responders was +117% (IQR +48%–+290%); the median ∆TMTV in responders was −88% (IQR −39%–−93%). Patients classified as non-responders (PD and SD) according to PERCIST 5 or imPERCIST5 criteria had increased ∆TMTV, except for two patients classified as SD for whom ∆TMTV had decreased by 50%. 

### 3.5. Survival Study

The median follow-up was 47.18 months. At the time of data analysis, 15 patients had died, and one patient was lost to follow-up (34 months after ICI treatment). Among them, 12 patients died of progressive disease, 1 of stroke (9 months after treatment initiation, classified as CR), 1 by accidental death (4.8 months after treatment initiation, classified as SD) and 1 for unknown cause (30.8 months after treatment initiation, classified as PR). Moreover, among the 14 patients still alive, nine had progressed.

The median PFS was 5.4 months, and the 2-year PFS rate was 20.7%. PFS was better in the responder group (CR and PR) versus non-responders (SD and PD), with a 2-year PFS rate of 54.5% vs. 0% (HR: 8.6, 95%IC: 2.7–27.4) (Supplementary Figure S3). PFS was higher in patients without new lesions than in patients with new lesions on evaluation PET/CT (*p* = 0.007) (Supplementary Figure S4).

**Figure 4:**
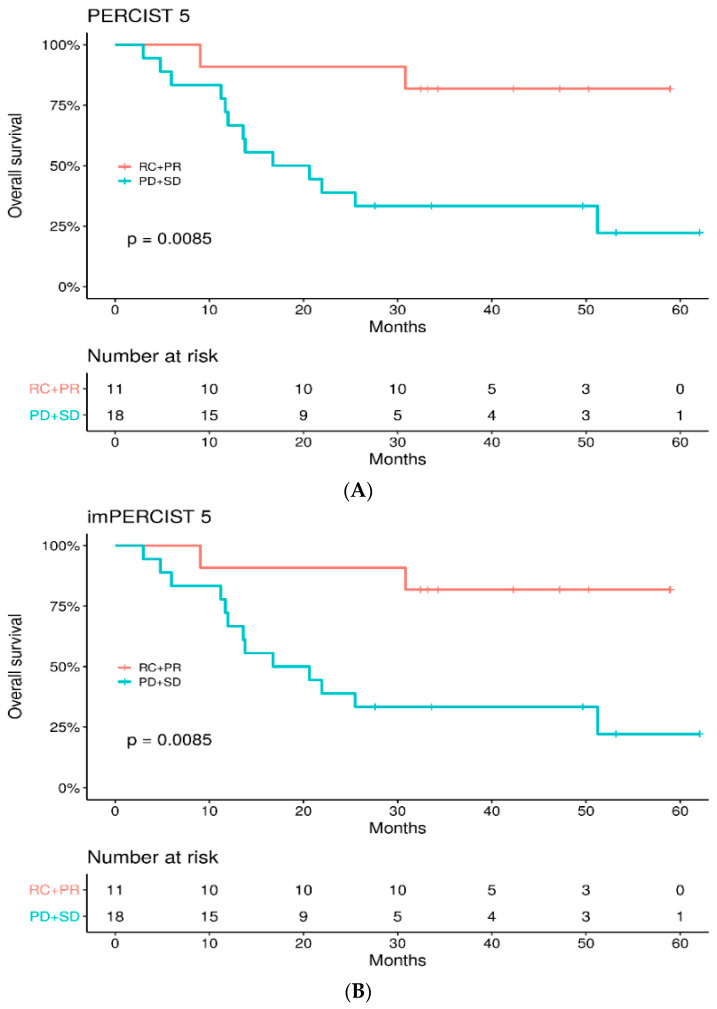
OS between responder (CR + PR) vs. non-responder (SD + PD) patients according to PERCIST5 (**A**) and imPERCIST 5 (**B**) criteria.

**Figure 5:**
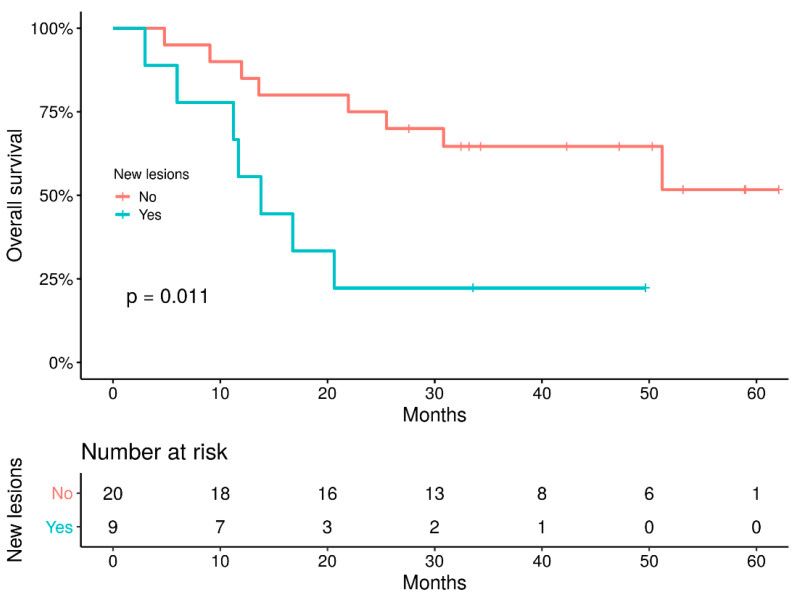
OS of patients with no new lesion versus patients with at least one new lesion.

The median OS was 51.2 months (IQR 13.6—not reached), and the 2-year OS rate was 58.6%. OS was significantly higher in the responder group versus non-responders using either PERCIST5 or imPERCIST5 criteria with a 2-year OS of 91% vs. 39%, respectively (HR: 5.96, 95%IC: 1.3–26.7) (Figure 4). Furthermore, OS was significantly betterer in patients without new lesions on the follow-up PET/CT (*p* = 0.011) (Figure 5).

**Figure 6:**
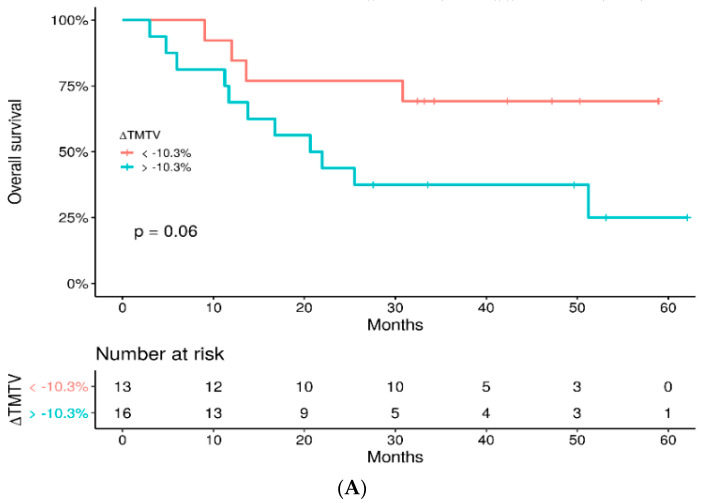

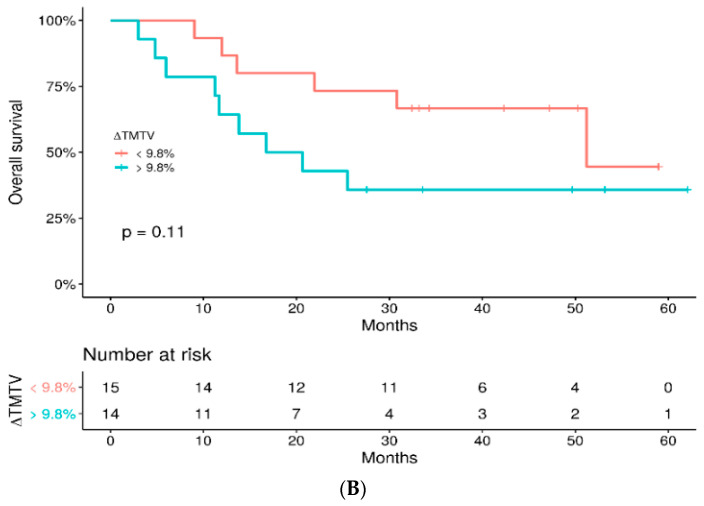
OS of patients with ∆TMTV less than −10.3% versus greater than −10.3% (**A**) and those with ∆TMTV less than +9.8% versus greater than +9.8% (**B**).

Complementary analysis survival was also performed according to thresholds for changes in TMTV at −10.3% (threshold obtained by ROC analysis) and at +9.8% (threshold corresponding to the median). On one hand, there was no significant difference in OS between groups, whatever cut-off was used (*p* = 0.14 and *p* = 0.093, respectively, Figure 6). On the other hand, PFS was longer in patients with the largest decrease in TMTV, with both cut-offs (*p* = 0.0011 and *p* < 0.001, respectively) (Supplementary Figure S5).

### 3.6. Patients’ Outcome

Twenty-eight patients had treatment discontinuation, for disease progression (*n* = 16, 57%), medical decision (mostly because of prolonged CR) (*n* = 4, 14%), toxicity of ICI (*n* = 5, 18%), premature death independent of disease (*n* = 2, 7%) and geriatric deterioration of general condition in one patient (4%).

The follow-up characteristics of the 11 patients classified as responders at 3 months (3 with RC + 8 with PR) are shown in Supplementary Table S1. Of these patients, nine were still alive at the end of the data collection, including five with a follow-up of more than 3 years. Of the eight patients initially classified as PR, five achieved a CR on follow-up PET/CT. Four of the initially responding patients had a disease recurrence: the first one had a brain recurrence occurring 2 months after initiation of immunotherapy, visualized on MRI but not on PET/CT. He was treated by cerebral radiotherapy and continued immunotherapy for some time. The second patient presented a progression of a renal lesion 6 months after the administration of a single course of immunotherapy (stopped for colitis) and could benefit from a local treatment by cryotherapy. The third one experienced progression with grelic lesions that were removed surgically, with no further treatment because of the patient’s age, and remained disease-free thereafter. The fourth one presented with a cutaneous recurrence, surgically removed, allowing a CR, and ICI was pursued.

A total of 22 patients developed disease progression, of whom 18 were classified as non-responders on the 3-month PET/CT, and four relapses occurred in initially responding patients. Five patients had elevated LDH levels at the time of recurrence or progression, and 14 had normal levels (data missing in three patients). Of these 22 patients, eight were still alive at the time of data analysis. Salvage treatments, such as chemotherapy, radiotherapy, surgery and targeted therapy, were introduced for patients who progressed on the 3-month PET assessment (Supplementary Table S2).

### 3.7. Assessment of Adverse Events

Twenty-two patients (76%) experienced adverse events from immunotherapy with grade 3 or 4 events in eight patients (evaluated with the CTCAE v4.0 scale). Five responding patients stopped the treatment prematurely for toxicity due to fibrosing pneumonitis, grade 3 colitis, colitis associated with skin toxicity (in 2 patients) and lipodystrophy-like skin toxicity.

The 3-month follow-up PET/CT revealed adverse events in four patients: colitis, thyroiditis, sarcoidosis associated with thyroiditis, and sarcoidosis with hypophysitis.

## 4. Discussion

In our study, the tumor response assessed at 3 months using imPERCIST 5 and PERCIST5 PET/CT criteria in patients with advanced or metastatic melanoma treated with first-line anti-PD1 +/−anti CTLA4 combination is a predictor of OS. The 2-year OS was 91% in the responder group versus 39% in the non-responder group, and the 2-year PFS was 54.5% versus 0%, with similar results according to PERCIST 5 or imPERCIST 5 criteria. Ito et al. [16], in 60 metastatic melanoma patients treated with ipilimumab, found that OS at 2 years was 61% in responders versus 33% in non-responders according to PERCIST5 criteria and 66% versus 29% according to imPERCIST5 criteria. Thus, similar to what we found, PERCIST5 and imPERCIST criteria yield both relevant results regarding patients’ outcomes. Iravani et al. evaluated the metabolic response with PERCIST criteria after 2 to 4 months of treatment with first-line ipilimumab/nivolumab combination in 31 patients and found CMR in 62% of patients, PMR in 19% and PD in 19% [25]. CMR at this first evaluation was associated with better OS than non-CMR response. Amrane et al. explored the value of different response criteria (RECIST, iRECIST, PERCIST and PECRIT) to evaluate response to immune checkpoint inhibitors as monotherapy, around 3 months for a first evaluation and sometimes later in the follow-up in 37 patients [13]. At the first evaluation, RECIST, iRECIST and PERCIST criteria seemed predictive of PFS and OS. Patients without response according to PERCIST criteria had a median PFS and OS of 6.1 and 11 months versus 23.8 and 26.1 months for those with a response. Likewise, in our cohort, responder patients did better regarding PFS and OS than non-responder patients. Of note, some of the initially responding patients eventually progressed, mostly in the oligometastatic mode, allowing local treatment and disease control.

The analysis of tumor metabolic volume has been evidenced as a relevant tool in the evaluation of metastatic melanoma treated by ICI, allowing not only baseline outcome prognostication [18,26] but also accurate prediction of the response to treatment [20,22,23]. In a study on 85 melanoma patients treated with ICI, the metabolic tumor volume at first evaluation proved to be an independent prognostic factor for OS, along with the presence of central nervous system lesions [20]. Annovazzi et al. also compared several PET parameters to assess response 3 to 4 months after either ipilimumab or PD-1 inhibitors as monotherapy in a total of 57 patients [22]. In the anti-PD1 group, PET parameters, such as EORTC, delta MTV and delta TLG, correlated with patients’ outcomes at one year in most of the patients with PD according to these PET metrics. In 36 patients evaluated between 2 to 4 months after treatment by pembrolizumab monotherapy, Vermeulen et al. found that PFS and OS were significantly longer in patients achieving a decreased/stable TMTV compared with those with increasing TMTV [23]. In our study, we found a similar result for PFS but not for OS, probably due to the limited size of our series of patients. The initial TMTV seems lower in our study than in other previously reported studies: 5.6 mL versus 6.8 mL to 28.2 mL [19,21,25,26]. This difference may be partly explained by the fact that the patients in our study were in first-line ICI and therefore possibly treated with less advanced disease.

In our study, one case of pseudoprogression was observed (3.5% of patients), and this is similar to the 4.7% incidence reported by Basler et al. in a series of 112 patients [27]. The incidence of pseudoprogression may be as high as 4–10% for patients with metastatic melanoma on immunotherapy [28]. However, as in other studies involving ICI, we observed that most patients with the appearance of new lesions had a worse outcome than patients with no new lesions at 3 months [15,16]. Anwar et al. reported that the number of new lesions but also their size predicted a patient’s outcome [15]. These results highlight the importance of taking into account all aspects of imaging (metabolism, metabolic volume, size and number of lesions). Moreover, the best response with ICI can be achieved beyond 3 months [25,29], emphasizing the need to accurately identify patients that would ultimately benefit from the treatment and those for whom an alternative should be considered in a timely manner. A potential benefit for the “stable disease” subcategory defined according to the new imPERCIST5 criteria would be to comfort the clinicians when the therapeutic strategy is to continue ICI despite progression according to the conventional PERCIST 5 criteria, if a patient’s clinical condition allows it [30], especially if the tumor volume remains low.

Retrospective data collection is one of the limitations of this study. Survival analysis was not totally independent of the response observed in the PET/CT evaluation. Indeed, PET/CT at 3 months was used for evaluation and therapeutic adaptation by hospital clinicians. Nevertheless, the follow-up evaluation of the patients was also guided by the clinical examination, the LDH level and the brain MRI, and decisions to continue or stop the immunotherapy were taken by an interdisciplinary committee based on a set of arguments, including the onset of serious adverse events. 

Another limitation of this study is the small number of patients recruited, which may have limited the power of the statistical analysis. This is due to our strict inclusion criteria: we chose to include patients with PET/CT performed on the same camera to limit variations in SUL potentially due to different acquisition or reconstruction methods between cameras.

The application of the PERCIST5 criteria is challenging. One difficulty is the target lesions’ size. Indeed, melanoma metastasis is sometimes small, and the use of SULpeak may underestimate the 18F-FDG uptake intensity of these small lesions. For three patients, target lesions were sub-centimetric and had a SULpeak below the PERCIST5 reference threshold but were clearly distinct from the surrounding background and were retained for analysis. 

At this time, there are no consensus criteria for the evaluation of immunotherapy with FDG PET/CT. However, it seems relevant to analyze several elements before classifying a patient as progressive, such as the time between the initiation of immunotherapy and the PET/CT evaluation, the ∆SULpeak, the ∆TMTV and the number of new lesions (if applicable). Even if FDG PET/CT occupies a growing role in oncology, the clinical and biological aspects remain essential for the evaluation and orientation of the therapeutic strategy. Physical evaluation, biological assessment with the long-established LDH in melanoma, but also the emerging circulating tumoral DNA [31] may help to put into perspective ambiguous metabolic response patterns.

## 5. Conclusions

The evaluation of tumor response with FDG PET/CT in patients with advanced or metastatic melanoma three months after first-line ICI using imPERCIST 5 and PERCIST5 criteria is significantly correlated with OS. Large prospective studies are needed to confirm these results and further evaluate the potential role of the total metabolic tumor volume.

## Figures and Tables

**Table 1:** Patient characteristics at ICI induction (WT for Wildtype; M for Mutated; NA for Missing Data).

Patient Characteristics	Patients nb (Total = 29)
Gender	
Female	14 (48%)
Male	15 (52%)
Type of treatment	
pembrolizumab	15 (52%)
nivolumab	9 (31%)
nivolumab + ipilimumab	5 (17%)
Breslow (mm)	
≤1	4
1, 1–4	9
>4	13
Missing data	3
Mutation status	
BRAF WT/M/NA	22/7/0
NRAS WT/M/NA	19/9/1
Ckit WT/M/NA	10/2/17
ECOG	
0	17
1	10
2	2
LDH level	
normal	19
high	8
Missing data	2
Type of primary melanoma	
cutaneous mucosal	23 4
unknown	2

**Table 2:** Correlation between PERCIST5 and imPERCIST5 criteria (PD: for progression disease, PR: for partial response, CR: for complete response and SD: for stable disease). k = 0.718.

	imPERCIST 5				
PERCIST 5	CR (*n*)	PR (*n*)	SD (*n*)	PD (*n*)	Total (*n*)
CR	3	0	0	0	3
PR	0	8	0	0	8
SD	0	0	4	0	4
PD	0	0	6	8	14
Total	3	8	10	8	29

## Data Availability

The data that support the findings of this study are available from the corresponding author upon reasonable request.

## Supplementary Materials

The following supporting information can be downloaded at: https://www.mdpi.com/article/10.3390/cancers14133190/s1, Table S1. Follow-up of responder patients; Table S2. Follow-up of non-responder patients according to PERCIST5 and imPERCIST5 criteria; Figure S1. Example of a partial response to ICI; Figure S2. Example of progression under ICI; Figure S3. PFS between responder (CR+PR) vs non-responder (SD+PD) patients according to PERCIST5 (Figure A) and imPERCIST 5 (Figure B) criteria; Figure S4. PFS of patients with no new lesion versus patients with at least one new lesion; Figure S5. PFS of patients with ∆TMTV less than −10.3% versus greater than −10.3% (Figure A) and those with ∆TMTV less than +9.8% versus greater than +9.8%.
