# Supplementary material for: 18FDG PET Assessment of Therapeutic Response in Patients with Advanced or Metastatic Melanoma Treated with First-Line Immune Checkpoint Inhibitors
Source: Cancers (Basel). 2022 Jun 29;14(13):3190. doi: 10.3390/cancers14133190 (PMC9264956; doi:10.3390/cancers14133190)

Supplementary Figure S1. Example of a partial response to ICI.

A: Maximum Intensity Projection at baseline showing multiple secondary lesions in lymph nodes (cervical, mediastinal, pararectal), and lungs; B: Maximum Intensity Projection at follow-up showing a major decrease in sum of SULpeak (-87%) and in TMTV (-76%).

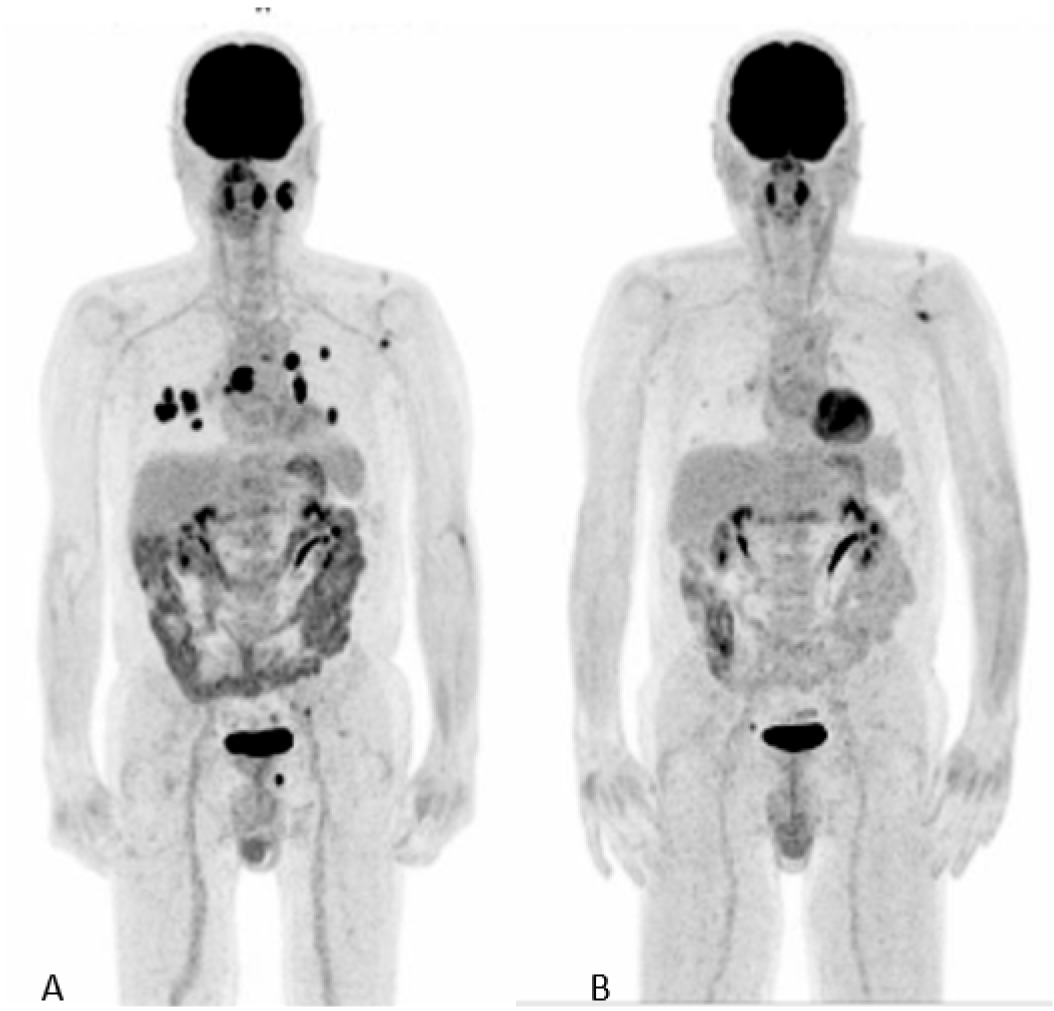

Supplementary Figure S2. Example of progression under ICI.

A: Maximum Intensity Projection at baseline showing multiple metastatic lesions with lymph node and bone involvement ; B: Maximum Intensity Projection at follow-up showing metabolic progression with PERCIST 5 with appearance of new lesions (in bone, lungs, liver and lymph nodes) and stability according to imPERCIST with an increase of ΔSUL peak increase not reaching progression cut-off (+19%) and a major increase of TMTV (+494%)

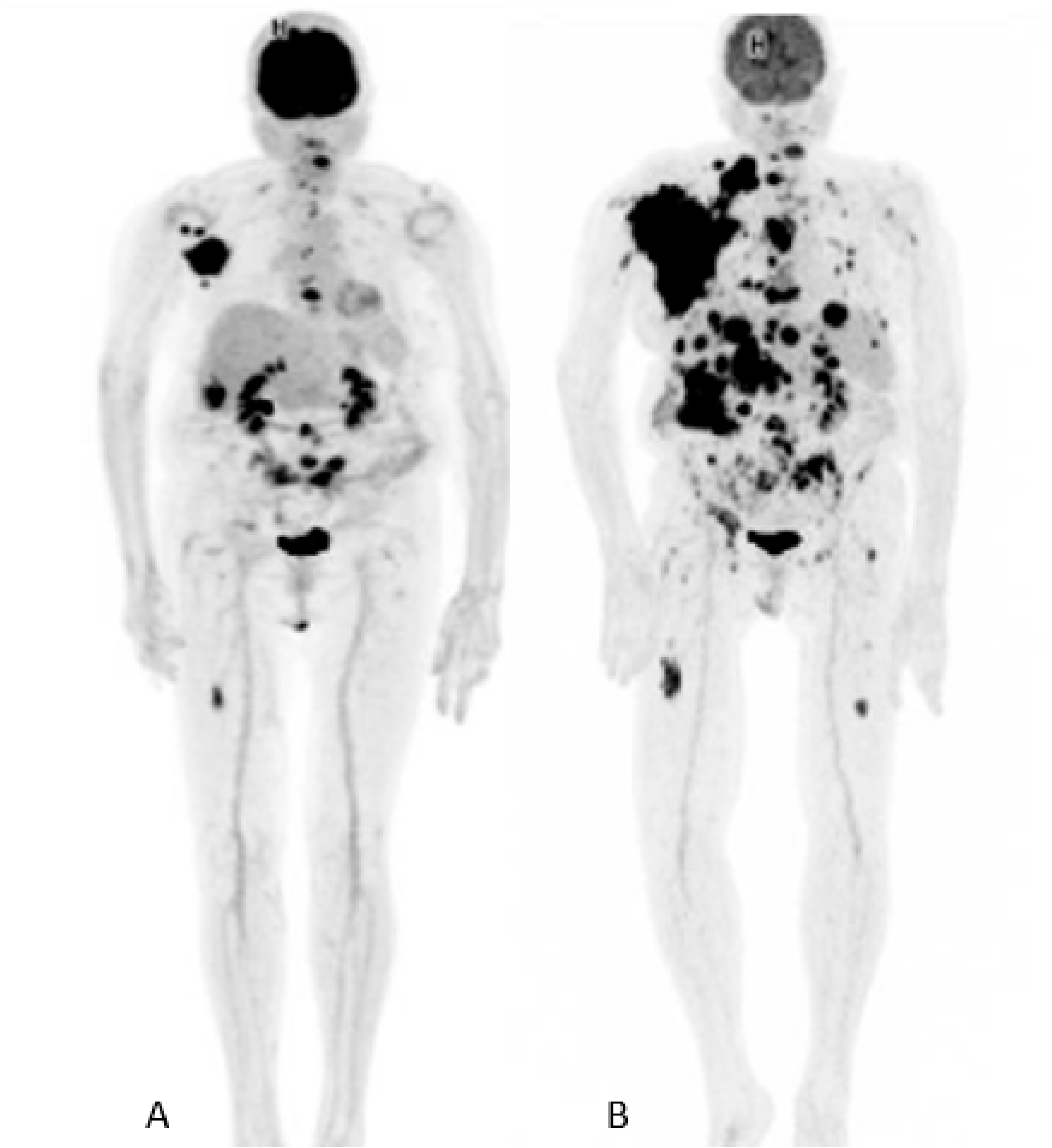

Supplementary Figure S3. PFS between responder (CR+PR) vs non-responder (SD+PD) patients according to PERCIST5 (Figure A) and imPERCIST 5 (Figure B) criteria.

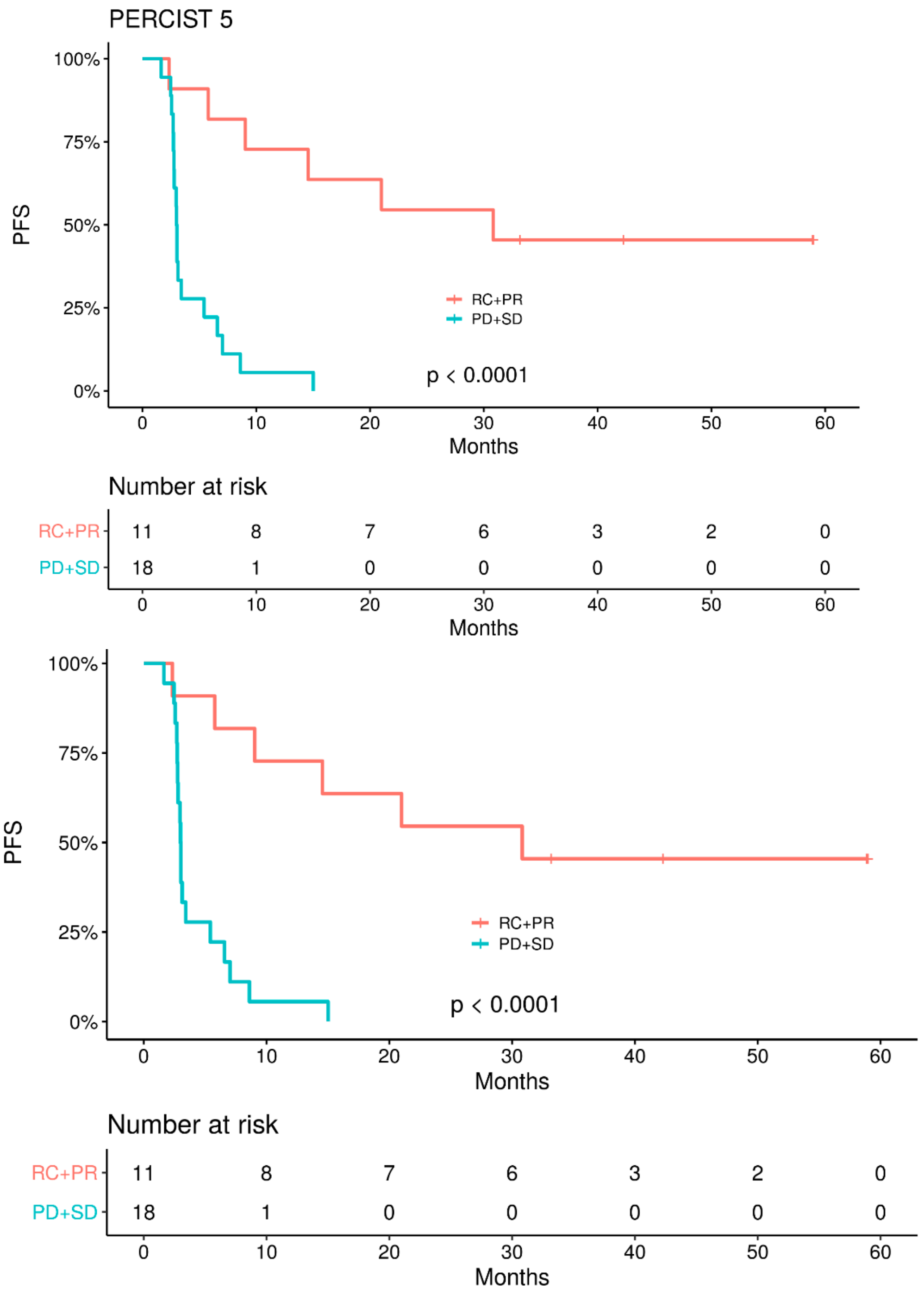

Supplementary Figure S4. PFS of patients with no new lesion versus patients with at least one new lesion.

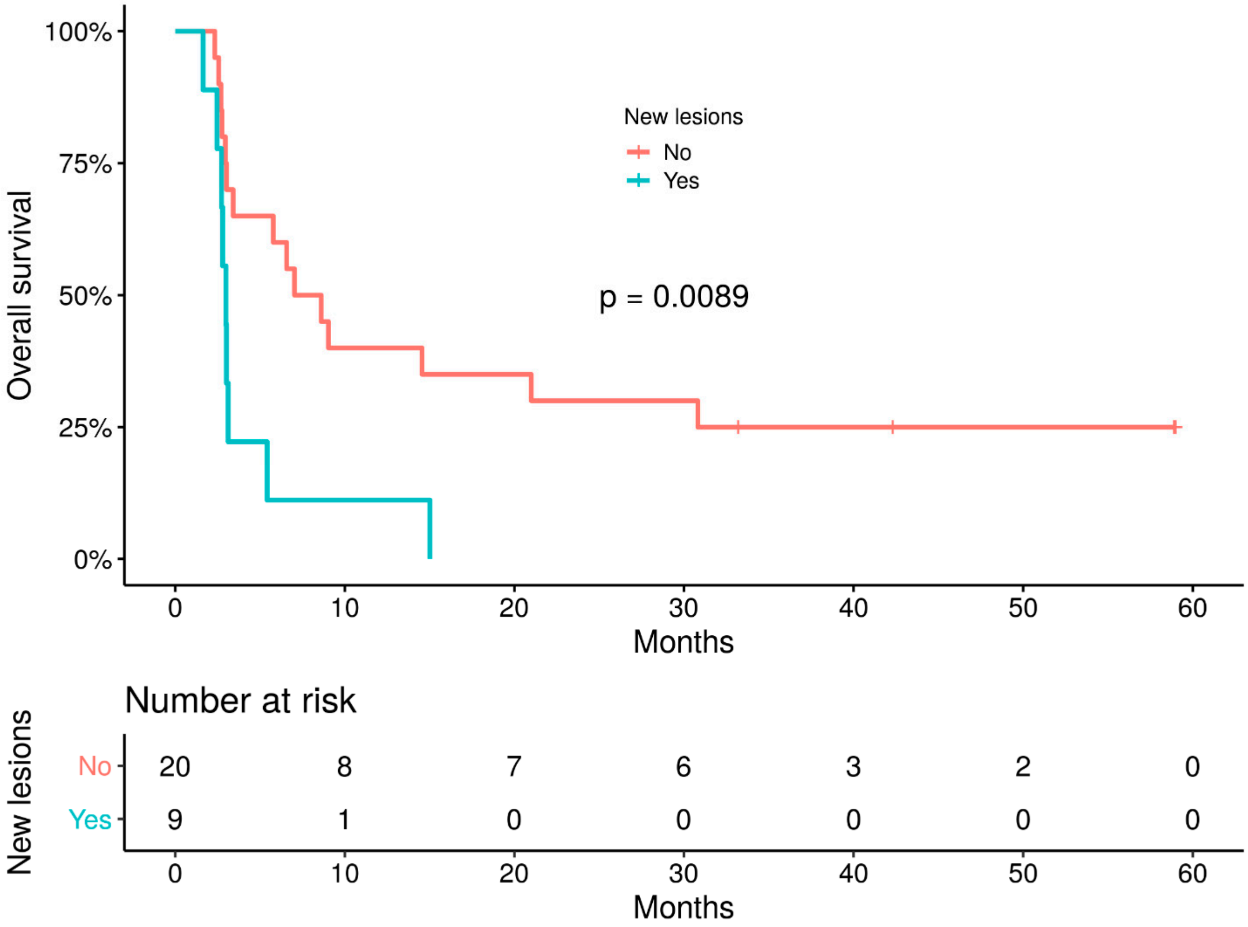

Supplementary Figure S5. PFS of patients with ΔTMTV less than -10.3% versus greater than -10.3% (Figure A) and those with ΔTMTV less than +9.8% versus greater than +9.8%

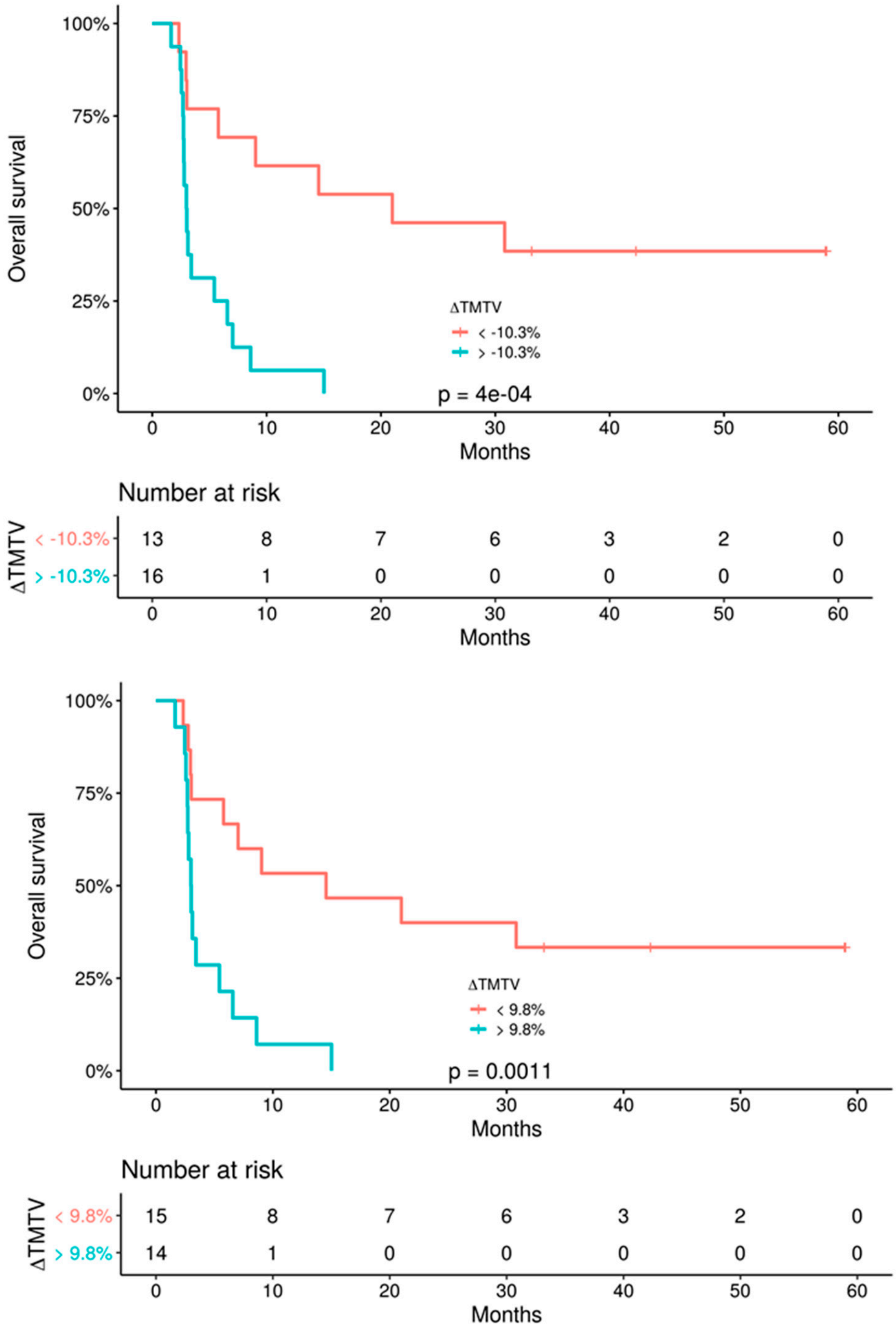

Supplement: Supplementary file 1 [file cancers-14-03190-s001.zip › cancers-1773131-supplementary figures.pdf]
